# Increase in ticagrelor use over time is associated with lower rates of ischemic stroke following myocardial infarction

**DOI:** 10.1186/s12872-019-1030-6

**Published:** 2019-03-04

**Authors:** Robin Henriksson, Anders Ulvenstam, Lars Söderström, Thomas Mooe

**Affiliations:** 1 Department of Public Health and Clinical Medicine, Umeå University, Umeå, Sweden; 2 Unit of Research, Education and Development, Region jämtland Härjedalen, Östersund, Sweden

**Keywords:** Ischemic stroke, Secondary prevention, Acute myocardial infarction, Antiplatelet therapy

## Abstract

**Objectives:**

To evaluate the impact of a rapid change in preferred treatment from clopidogrel to ticagrelor on the risk of ischemic stroke following acute myocardial infarction (AMI).

**Methods:**

Data for AMI patients treated with either clopidogrel or ticagrelor were obtained from the Swedish Register of Information and Knowledge about Swedish Heart Intensive Care Admissions (RIKS-HIA). Patients were divided into two cohorts, each covering a two-year time period; the initial prescription of ticagrelor (20 Dec 2011) was used as a cut-off point. Patients in the early cohort (*n* = 23,447) were treated with clopidogrel, while those in the later cohort (*n* = 24,227), were treated with either clopidogrel (47.9%) or ticagrelor (52.1%). Kaplan-Meier analyses were used to assess the risk of ischemic stroke over time, with multivariable Cox regression analyses used to identify predictors of ischemic stroke.

**Results:**

Of 47,674 patients, there were 1203 cases of ischemic stroke. Cumulative Kaplan-Meier incidence estimates of ischemic stroke after one year were 2.8% vs. 2.4% for the early and late cohorts, respectively (*p* = 0.001). Older age, hypertension, diabetes, previous stroke, congestive heart failure, atrial fibrillation, and ST-elevation myocardial infarction were associated with an increased risk of ischemic stroke. Percutaneous coronary intervention and statins at discharge were associated with a decreased risk of ischemic stroke, as was higher estimated glomerular filtration rate. Membership of the late cohort correlated with a 13% reduction in the relative risk of ischemic stroke.

**Conclusions:**

The introduction of ticagrelor as well as an improved management of AMI was associated with a lower rate of ischemic stroke in a relatively unselected AMI population.

**Electronic supplementary material:**

The online version of this article (10.1186/s12872-019-1030-6) contains supplementary material, which is available to authorized users.

## Introduction

Ischemic stroke following acute myocardial infarction (AMI) is a known and feared complication with potentially devastating consequences. The reported incidence of ischemic stroke post AMI varies from 2.1% to 4.1% after 1 year [1]. Myocardial infarction (MI) complicated by ischemic stroke incurs a high societal burden and increased morbidity. Furthermore, studies have shown a significant increase in mortality associated with this complication [2]. In a study that compared mortality rates in AMI patients with and without ischemic stroke, an increase of between 16% and 19% in absolute mortality rate (during the first year) was seen in those patients with AMI complicated by ischemic stroke. In total, the mortality rate was 36.5% after 1 year in patients suffering an ischemic stroke after AMI [3]. Currently, dual antiplatelet therapy (DAPT) [4, 5] is an essential component of treatment and secondary prevention, irrespective of whether the patient suffering from an acute coronary syndrome (ACS) is treated invasively with percutaneous coronary intervention (PCI) or not. DAPT involves the combination of a P2Y12-inhibitor and aspirin. Clopidogrel, the favoured P2Y12-inhibitor since 2001, is now being superseded by ticagrelor, a faster and more potent oral (reversible) P2Y12-inhibitor [6, 7]. The PLATelet inhibition and patients Outcomes (PLATO) trial [8] compared clopidogrel with ticagrelor in patients presenting with acute coronary syndrome. The PLATO trial overall demonstrated the superiority of ticagrelor, however, at the twelve month mark no difference in the incidence of ischemic stroke following AMI was found (1.1% vs. 1.1%). Compared to a real world population, patients in the PLATO trial were younger, had less co-morbidity and were at lower risk for ischemic stroke. Based on the overall risk factor profile, and prognoses of the patient population in the PLATO trial, it can be concluded that the external validity of that trial may be limited, regarding the risk of ischemic stroke. Consequently, there is a knowledge gap as to the effect of ticagrelor on the risk of ischemic stroke in an unselected patient population with AMI. We therefore aimed to investigate if the change in preferred treatment from clopidogrel to ticagrelor would alter the risk of ischemic stroke in a more representative AMI population.

## Methods

**Fig. 1:**
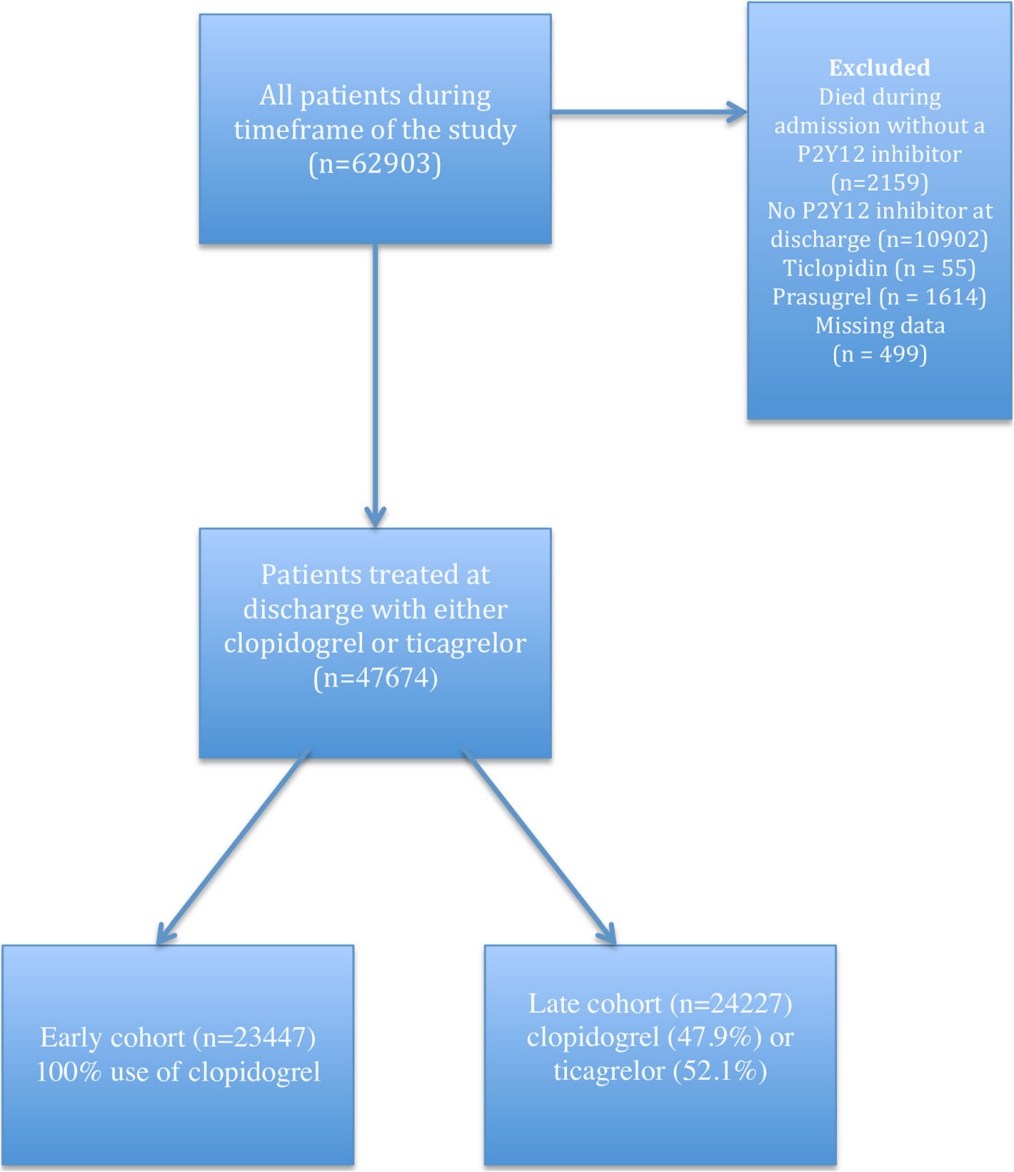
Study flow chart

The study population was obtained from The Swedish Web-system for Enhancement and Development of Evidence-based care in Heart disease Evaluated According to Recommended Therapies (SWEDEHEART). SWEDEHEART is a national quality register, launched in 2009, following the merger of several registers including the Register of Information and Knowledge about Swedish Heart Intensive Care Admissions (RIKS-HIA). Details about this register have been described elsewhere [9]. Data is available from SWEDEHEART upon request and with granted ethics approval. Patients presenting with ACS and hospitalized in a coronary care unit (CCU) are included in the RIKS-HIA-register. All participants in this study had been diagnosed with AMI [10]. Patient selection was based on admittance between the 8^th^ of December 2009 and 31^st^ of December 2013 and treatment with either clopidogrel or ticagrelor at discharge. Patients discharged without or with other P2Y12 inhibitors were excluded, as were patients that died during admission, not yet initiated on P2Y12 inhibitors as shown in Fig. 1.

Routinely collected patient information includes demographics, medical history, risk factors, biochemical markers, diagnoses, interventions, and medications. These data are collected on admission, during hospitalization, and at discharge. The RIKS-HIA register was then combined with the National Patient Register (NPR) and the Cause of death register in order to identify patients with ischemic stroke as well as date of death in applicable cases. The diagnosis codes I63 and I64, from *the International Classification of Diseases-Tenth Revision,* were used to identify patients suffering ischemic stroke. The NPR has been validated, with sensitivity and positive predictive values of a diagnosis of stroke found to be approximately 90% [11]. The registers are based on the Swedish population with no exclusion criteria. All Swedish citizens have a unique personal identification number which enables follow-up in different registers. This means that loss of follow-up is limited to patients emigrating from Sweden. With an average emigration rate of approximately 0.5% during the study period this likely did not affect the results of the study. Several register variables were redefined prior to data analysis. The variable “smoking” was defined as smoking during the past month. “Atrial fibrillation” was defined as previous atrial fibrillation or atrial fibrillation during hospitalization. “Heart failure during hospitalization” includes patients receiving either treatment with intravenous diuretics, or patients with clinical signs of heart failure (e.g. pulmonary rales during hospitalization). The variable eGFR was calculated using the Chronic Kidney Disease Epidemiology Collaboration equation [12] (CDK-EPI) and for practical reasons all patients were assumed to be Caucasian. We estimated the treatment length of clopidogrel and ticagrelor based on dispensed prescriptions from a pharmacy by combining our data with data from the Swedish prescribed drug register.

### Statistical analysis

**Fig. 2:**
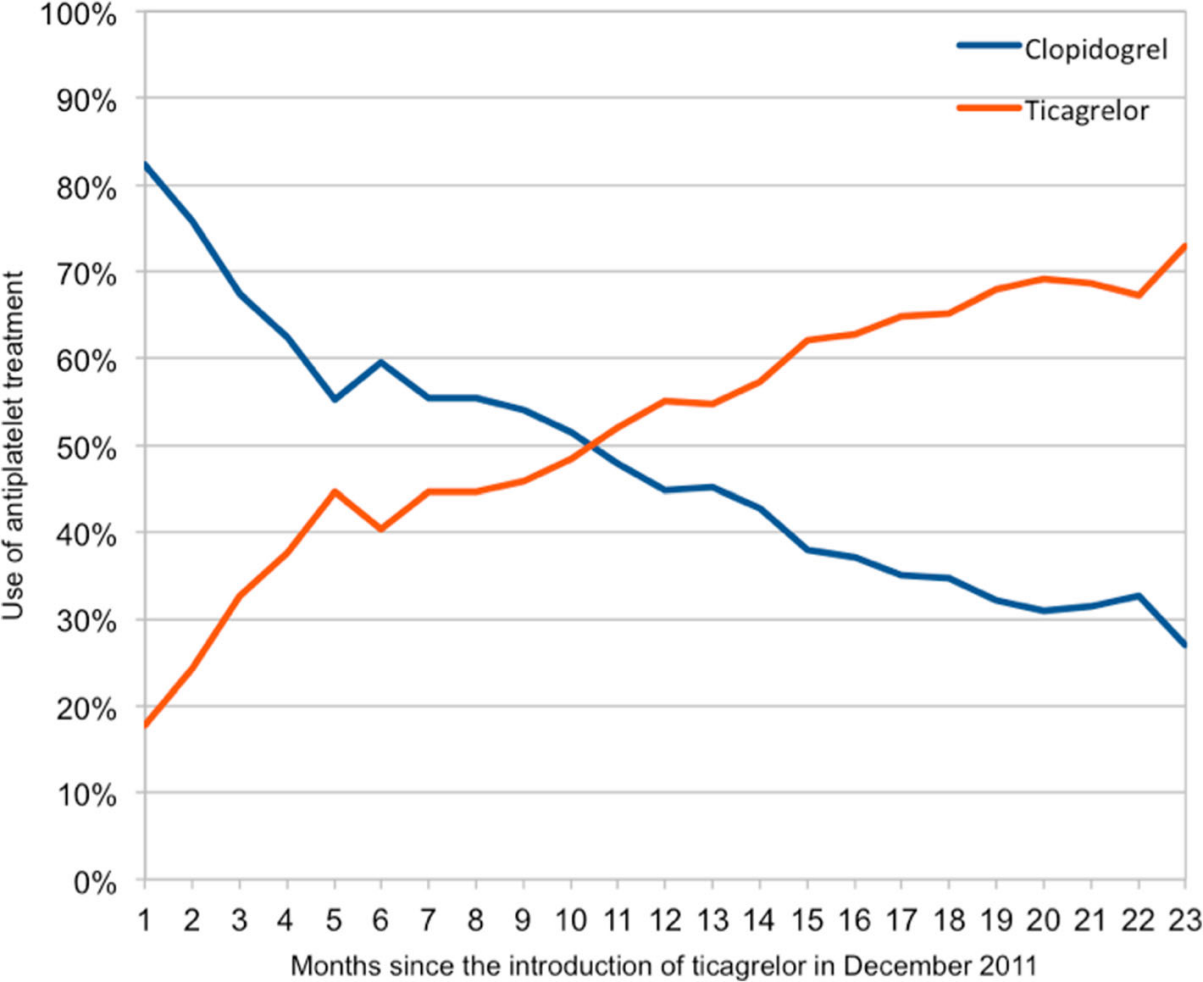
The rapid change in preferred discharge treatment between clopidogrel and ticagrelor

Using the first prescription of ticagrelor as a cut-off point (20 December 2011), the study period was divided into two time periods of similar length to create two cohorts. The first period (early cohort) ran from the 8th December 2009 through 19th December 2011. The second, late cohort ran from the 20th December 2011 until 31st December 2013, when the register follow-up ended. All patients in the early cohort were treated exclusively with clopidogrel, whereas either clopidogrel or ticagrelor was administered to the late cohort; the use of ticagrelor increased gradually during the study period (Fig. 2). This model was chosen to avoid any selection bias (that could arise when directly comparing the treatments) given that ticagrelor was the preferred option for low-risk patients when it was introduced in routine care. Thus, in the present analysis we compared all patients registered during the early period (clopidogrel only) with all patients registered during the late period (increased use of ticagrelor). To further analyze outcomes based on the proportion of patients receiving ticagrelor, a sub analysis was made in the late cohort. Patients were grouped based on admittance, and the percentage of ticagrelor use was calculated: one group for the first third of the period, the other group for the remaining two thirds, and these two groups were then compared to the early cohort. Sensitivity analyses were also performed based on matching and duration of prescription of P2Y12- inhibitor. In order to get a representative study population, there were no exclusion criteria in regard to co-morbidities or age. Patients that died during admission, were discharged without a P2Y12-inhibitor, or were treated with prasugrel or ticlopidin were excluded as shown in Fig. 1.

Statistical results are presented as median with the 25^th^ and 75^th^ percentile for the continuous variables and as percentages for categorical variables. Comparisons were made between the early and late cohorts on baseline characteristics as well as medication at admittance and discharge**.** Comparisons were also made between patients discharged with clopidogrel vs. ticagrelor in the late cohort. The Mann-Whitney U-test was used for the continuous variables and the Pearson Chi-square test was used for categorical variables. Kaplan-Meier analyses were used to estimate and visualize the risk of ischemic stroke after AMI for the early and late cohorts, and in sub-analyses comparing the early cohort and sub groups of the late cohort, using the log-rank test. We also measured the unadjusted incidence rate at 3-month intervals in the two cohorts. A *p*-value < 0.05 was considered significant. Univariable and multivariable Cox proportional hazard models were used to identify predictors of ischemic stroke during the study period. Variables included in the multivariable Cox regression analysis had either been previously described as a risk factor, were deemed clinically relevant, or predicted risk in a univariate analysis (*P* < 0.1). This combination of both a numerical and a clinical method of variable selection were chosen to include all available variables of potential importance. Non-significant variables were stepwise excluded, according to their level of significance during iterative multivariable Cox regression analyses, until reaching a final multivariable Cox regression model. The final multivariable Cox regression analysis was based on complete cases and we accepted missing values up to 10%. A corresponding Cox analysis including ticagrelor vs. clopidogrel as predictor variable was performed within the late cohort. Results are presented as hazard ratios with a 95% confidence interval. All statistical analyses were performed using IBM SPSS v.24 with the exception of the matched analysis in the Additional file 1, which was made using the “Matchit” software in the statistical package “R”.

## Results

In total, 47674 study participants with AMI were included, with 23447 in the early clopidogrel-only cohort, and 24227 patients in the late cohort (47.9% treated with clopidogrel vs. 52.1% with ticagrelor).

### Baseline characteristics

The baseline cohort characteristics are shown in Table 1. While demographic variables of age and female sex in the early vs. late cohort were comparable, the proportion of smokers was significantly higher in the early cohort. Several comorbidities (i.e. diabetes, atrial fibrillation, ST-elevation myocardial infarction, previous ischemic stroke, intra-cerebral hemorrhage, or previous dialysis) also showed no significant differences when comparing the early vs. late cohort. Estimated glomerular filtration rate (eGFR) was also comparable across both cohorts. Peripheral artery disease and hypertension were more common (significantly) in the late cohort. Conversely, this cohort had significantly lower rates of heart failure during hospitalization, and previous MI. Regarding reperfusion strategy, a significantly higher proportion of patients in the late cohort underwent PCI as well as CABG surgery during hospitalization, with the use of thrombolysis during hospitalization significantly lower.

**Table 1:** Baseline characteristics and medication at discharge, early vs. late cohort

	Early (*n* = 23,447)	Late (*n* = 24,227)	*P*-value
Age (median)	70 (61–79)	70 (61–79)	0.08
Women%	33.5	33.7	0.89
Smoking%	23.1	22.2	0.02
Diabetes%	21.7	22.3	0.12
STEMI^a^%	36.9	37	0.9
Hypertension%	53	54.6	<0.001
Atrial fibrillation%	13.9	14.1	0.38
Heart failure during hospitalization%	20.2	18.9	<0.001
Previous MI^b^%	8.5	7.3	< 0.001
Previous ischemic stroke%	7.0	6.8	0.42
Previous hemorrhagic stroke%	1.1	1.3	0.08
Previous dialysis%	0.5	0.5	0.96
Previous PAD^c^%	4.4	4.9	0.03
Thrombolysis during hospitalization%	1.7	1.2	<0.001
PCI^d^ during hospitalization%	71.1	75.4	<0.001
CABG^e^ during hospitalization%	1.1	1.4	< 0.001
eGFR^f^	77.7 (60–90.8)	78.1 (60.2–91.2)	0.06
Aspirin	97	95.6	<0.001
ACEI/ARB^g^	78.6	79.8	<0.01
Statins	91.1	91.5	0.14
Oral anticoagulants	4.5	6.3	<0.001
Beta blockers	90.5	89.1	<0.001
Calcium inhibitors	15.3	16.2	0.01
Diuretics	26.4	23.5	<0.001

^a^ST-elevation myocardial Infarction

^b^Myocardial infarction

^c^Peripheral artery disease

^d^Percutaneous coronary intervention

^e^Coronary artery bypass graft surgery

^f^Estimated glomerular filtration rate

^g^*ACEI* Angiotensin converting enzyme-inhibitors, *ARB* Angiotensin receptor blockers

### Medication

At discharge, patients were, overall, well medicated and treated according to ESC (European society of cardiology) guidelines. Table 1 shows that, vs. the early cohort, more patients in the late cohort were treated with ACE-inhibitors/angiotensin receptor blockers, oral anticoagulants, and calcium channel inhibitors; fewer were prescribed aspirin, beta-blockers, and diuretics. Rates of statin usage were comparable. Medication at admission is shown in the Additional file 1: Table I. On admission, patients in the early cohort were more likely to use aspirin and diuretics, and less likely to use ACE-inhibitors/angiotensin receptor blockers, calcium channel inhibitors and oral anticoagulants than the late cohort. The use of statins and beta-blockers were comparable for both cohorts

### Incidence of ischemic stroke

**Fig. 3:**
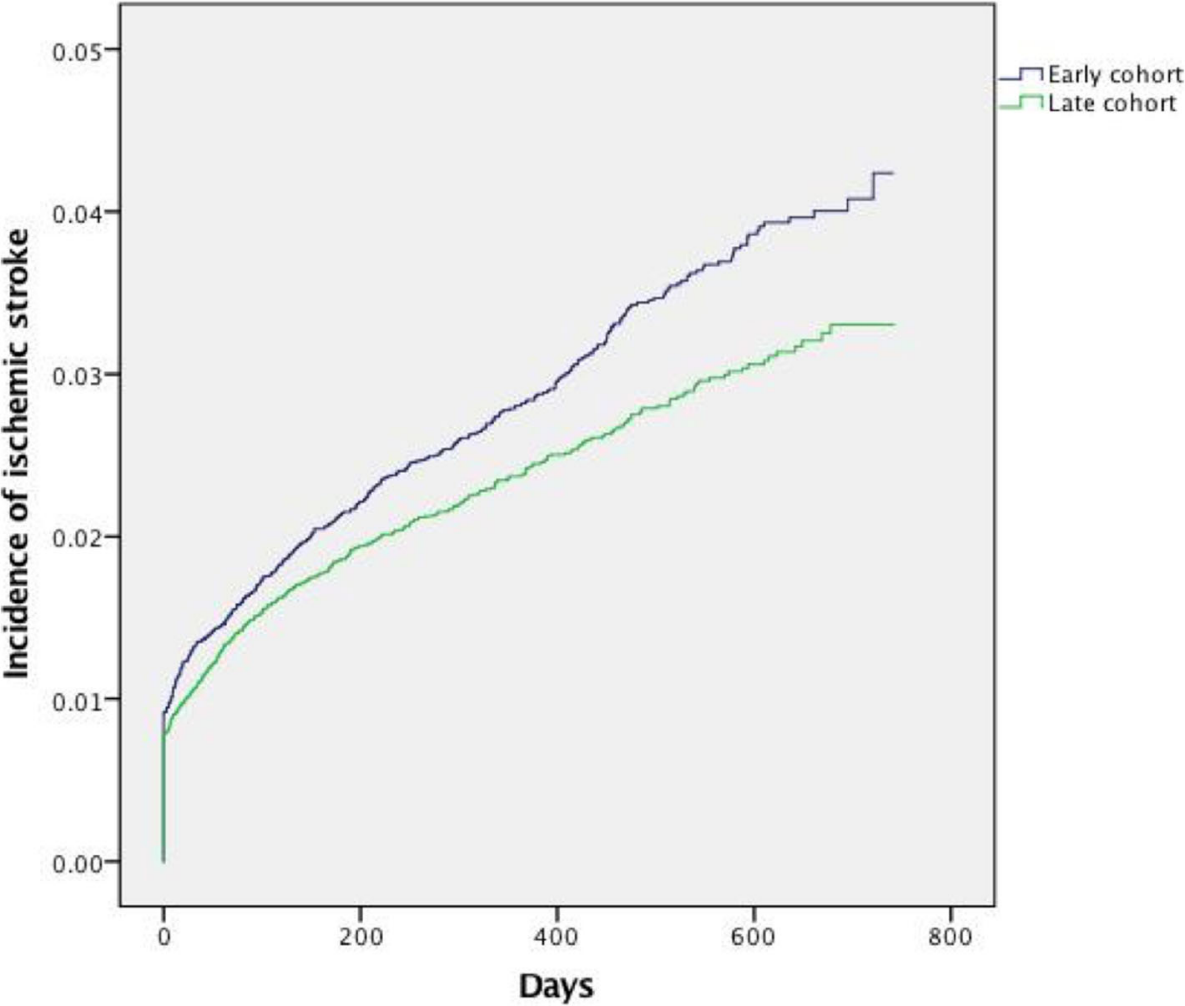
Kaplan-Meier estimates of the incidence of ischemic stroke among 47,674 AMI patients in the early (*n* = 23,447) vs. late (*n* = 24,227) cohort

Of 47674 patients, there were 1203 cases of ischemic stroke. Cumulative Kaplan-Meier incidence estimates of ischemic stroke after one year were 2.8% in the early cohort versus 2.4% in the late (p=0.001 log-rank test), giving a relative risk reduction of 14.3% (Fig. 3). Table 2 shows unadjusted incidence rates of ischemic stroke at 1, 3,6, 9, 12 and 15 months.

**Table 2:** Unadjusted incidence rates of ischemic stroke

Early cohort		Late cohort	
1 month	1.3%	1 month	1.05%
3 months	1.6%	3 months	1.43%
6 months	1.98%	6 months	1.72%
9 months	2.22%	9 months	1.9%
12 months	2.39%	12 months	2.05%
15 months	2.6%	15 months	2.15%

### Predictors of ischemic stroke

Results of the final multivariable Cox regression analysis are shown in Table 3. The univariable Cox regression analysis and the first run of the multivariable Cox regression analysis are shown in tables II and III in the Additional file 1. Older age, hypertension, diabetes, ST-elevation MI, previous ischemic or hemorrhagic stroke, signs of congestive heart failure during hospitalization, and atrial fibrillation were associated with an increased risk of ischemic stroke in the multivariable Cox model. Treatment with PCI, statins at discharge, higher eGFR and membership of the late cohort were independently associated with a lower risk of ischemic stroke. The number of dispensed prescriptions (a surrogate for treatment length) was found to be insignificant in the multivariable Cox regression model. However, membership of the late cohort was associated with a 13% reduction in the relative hazard of ischemic stroke.

**Table 3:** Predictors of ischemic stroke in a multivariable Cox regression model (*n* = 42,478)

Variable	HR (95% CI)	*P*-value
Age 65–74 years^a^	1.65 (1.34–2.03)	< 0.001
Age ≥ 75 years	2.18 (1.78–2.68)	< 0.001
STEMI^b^	1.27 (1.11–1.46)	< 0.001
Diabetes	1.23 (1.07–1.41)	< 0.01
Hypertension	1.17 (1.02–1.35)	0.03
Atrial fibrillation	1.92 (1.68–2.2)	< 0.001
Heart failure during hospitalization	1.29 (1.27–1.48)	< 0.001
PCI^c^ during hospitalization	0.72 (0.63–0.83)	< 0.001
CABG^d^ during hospitalization	1.93 (1.28–2.9)	< 0.01
Previous ischemic stroke	3.12 (2.7–3.62)	< 0.001
Previous hemorrhagic stroke	1.54 (1.07–2.21)	0.02
Statins at discharge	0.8 (0.67–0.94)	0.01
eGFR^e^	0.996 (0.993–0.999)	0.01
Belonging to the late cohort	0.87 (0.77–0.99)	0.03

^a^Age ≤ 64 years as reference for the different age categories

^b^ST-elevation myocardial infarction

^c^Percutaneous coronary intervention

^d^Coronary artery bypass graft surgery

^e^Estimated glomerular filtration rate

### Sub-analyses within the late cohort

As displayed in Table 4, there were significant differences between patients discharged with clopidogrel vs. those discharged with ticagrelor on all variables except thrombolysis during hospitalisation. Overall patients treated with ticagrelor were younger, had less co-morbidity, presented with STEMI and were treated with PCI to a greater extent. A multivariable Cox regression analysis within the late cohort including the variable ticagrelor at discharge is shown in Table 5. Older age, STEMI, diabetes, hypertension, previous ischemic stroke, atrial fibrillation and heart failure during hospitalization were associated with an increase in the hazard ratio of ischemic stroke, while PCI during hospitalization, statins and ticagrelor at discharge predicted a lower risk of ischemic stroke.

**Table 4:** Baseline characteristics within the late cohort. Patients discharged with clopidogrel vs. ticagrelor

	Clopidogrel (*n* = 11,597)	Ticagrelor (*n* = 12,632)	*P*-value
Age (mean)	72.9	66.7	<0.001
Women%	38.4	29.5	<0.001
Smoking%	18	26.1	<0.001
Diabetes%	24.5	20.3	<0.001
STEMI^a^%	26.4	46.8	<0.001
Hypertension%	60.2	49.4	<0.001
Atrial fibrillation%	20.4	8.4	<0.001
Heart failure during hospitalisation%	22.8	15.3	<0.001
Previous MI^b^%	10.1	4.7	<0.001
Previous ischemic stroke%	9.4	4.4	<0.001
Previous hemorrhagic stroke%	1.7	1.1	<0.001
Previous dialysis%	0.8	0.3	<0.001
Previous PAD^c^%	6.5	3.4	<0.001
Thrombolysis during hospitalisation%	1.3	1.1	0.21
PCI^d^ during hospitalisation%	61.9	87.7	<0.001
CABG^e^ during hospitalisation%	1.7	1.2	<0.01
eGFR^f^ (mean)	70.5	78.53	<0.001

^a^ST-elevation myocardial Infarction

^b^Myocardial infarction

^c^Peripheral artery disease

^d^Percutaneous coronary intervention

^e^Coronary artery bypass graft surgery

^f^Estimated glomerular filtration rate

**Table 5:** Predictors of ischemic stroke in a multivariable Cox regression model within the late cohort (*n* = 24,227)

Variable	HR (95% CI)	*P*-value
Age 65–74 years^a^	2.16 (1.49–2.72)	<0.001
Age ≥ 75 years	2.61 (1.96–3.49)	<0.001
STEMI^b^	1.45 (1.2–1.76)	<0.001
Diabetes	1.24 (1.03–1.5)	0.027
Hypertension	1.27 (1.04–1.55)	0.021
Atrial fibrillation	1.88 (1.55–2.27)	<0.001
Heart failure during hospitalization	1.24 (1.03–1.55)	0.024
PCI^c^ during hospitalization	0.68 (0.56–0.84)	<0.001
Previous ischemic stroke	2.81 (2.29–3.5)	<0.001
Statins at discharge	0.64 (0.51–0.8)	<0.001
Ticagrelor at discharge	0.77 (0.63–0.94)	<0.01

^a^Age ≤ 64 years as reference for the different age categories

^b^ST-elevation myocardial infarction

^c^Percutaneous coronary intervention

To further evaluate the impact of the change in preferred treatment from clopidogrel to ticagrelor in terms of the risk of ischemic stroke, we compared Kaplan-Meier estimates of the incidence of ischemic stroke in the early cohort vs. the corresponding estimates in first third of the late cohort (Additional file 1: Figure I), in which 34.6% of patients used ticagrelor and 65.4% used clopidogrel. Correspondingly, in a second analysis we compared the early cohort vs. the last two thirds of the late cohort (Additional file 1: Figure II). Use of ticagrelor rose to 61.1% in the last two thirds of the late cohort, with the use of clopidogrel use falling to 38.9%. Due to a shorter follow-up, and fewer patients in the subgroups of the late cohort, estimated incidence data primarily illustrate a trend towards fewer cases of ischemic stroke among patients treated with ticagrelor vs. clopidogrel at 1 year. The incidence of ischemic stroke in the early cohort was 2.8% compared to 2.5% in the first third, and 2.3% in the last two thirds of the late cohort, respectively.

### Matched analysis

We also performed a matched analysis based on the most important risk factors from the Cox analysis: age, previous ischemic stroke, CABG during hospitalization and atrial fibrillation. Based on 25,262 matched patients and 455 events of ischemic stroke, the relative risk reduction of ischemic stroke in the late cohort was 35%, using Kaplan-Meier analysis.

### Prescription patterns for clopidogrel and ticagrelor in the early vs. late cohort

As shown in Table 6 there was a clear trend towards more dispensed prescriptions from a pharmacy in the late cohort. In the early cohort, 44.6% of the patients had four or more dispensed prescriptions, which roughly approximates to one year of treatment. This figure increased to 50.4% and 72.3% for the clopidogrel and ticagrelor groups in the late cohort, respectively, translating into longer treatment durations.

**Table 6:** Prescriptions dispensed in the early and late cohort, based on medication

N of dispensed prescriptions	Early cohort (% (*n* = 23,447))	Late cohort-clopidogrel (% (*n* = 10,396))	Late cohort-ticagrelor (% (*n* = 11,897))
1	26.7	22.8	9
2	14.1	10.1	6.9
3	6.4	6.4	5.9
4	44.6	50.4	72.3
Excluded due to drug switch	8.3	10.4	5.8

### Incidence of ischemic stroke in patients with at least one year of P2Y12-inhibitor treatment

An additional sensitivity analysis was made including only patients with four or more prescriptions, and the Kaplan-Meier incidence estimates of ischemic stroke was 2.2% in the early cohort, compared to 1.5% in the late cohort (*p* < 0.001 log-rank test).

## Discussion

Randomized clinical trials (RCTs) are fundamental in establishing the effect of a given treatment. However, despite being the gold standard, the results of RCTs are seldom directly applicable to a heterogeneous (i.e. general) population. By observing the impact of treatments in a real life setting, their effects in clinical practice can be predicted. This is one of the first large-scale studies to use a relatively unselected AMI population in evaluating the impact of ticagrelor on the risk of ischemic stroke following AMI. The present study shows that the increased adoption of ticagrelor over clopidogrel is associated with lower rates of ischemic stroke in a fairly unselected AMI population. The two cohorts used in this study have comparable characteristics, but differ in some respects. Due to the large sample size, numerically small differences may result in statistically significant data. The increased use of PCI in the late cohort, even though significantly associated with lower rates of ischemic stroke post MI, does not fully explain the overall difference between the two cohorts, as it is only one of several factors that are associated with a reduction of ischemic stroke. The only medication type that predicted a statistically significant decrease in the risk of ischemic stroke at discharge was statins, and these were used to a comparable degree across cohorts. However, in the late cohort, more patients were treated with ACE-inhibitors/angiotensin receptor blockers, calcium channel blockers and oral anticoagulants (Table 2), which could suggest an overall better management of hypertension, heart failure and atrial fibrillation. This may translate into a lower risk of ischemic stroke although the individual drug did not predict a lower risk in the multivariable Cox regression analyses (Additional file 1: Table III, and Table 3). The reduction of ischemic stroke in the late cohort, in which the majority of patients received ticagrelor, contradicts data reported in the PLATO-trial [8], as well as a published sub-group analysis looking at ACS patients with a planned invasive strategy [13]. In those two studies, no differences in the incidence of ischemic stroke between patient-groups treated with clopidogrel vs. ticagrelor were found. A second PLATO sub-group analysis investigating NSTE-ACS patients with and without revascularization also found no significant difference in the incidence of total stroke [14]. A third sub-group analysis, based on the elderly PLATO-population, found a numerically, albeit statistically insignificant increase in stroke events in the ticagrelor vs. clopidogrel treated group [15]. Problematically, the last two studies failed to distinguish between the different types of stroke, which could obscure a preventative effect on ischemic stroke due to an increase in hemorrhagic stroke. A meta-analysis comparing clopidogrel to newer oral and intravenous P2Y12-inhibitors (including, but not restricted to, ticagrelor) reported similar results, with no significant difference in the rate of stroke events [16]. The incidence of ischemic stroke after AMI in studies comparing clopidogrel with ticagrelor have ranged from 0.9% [13] to 1.1% [8] at 1 year, compared with the incidences that we now report of 2.8% vs. 2.4% in the early vs. late cohorts, respectively. Previous comparisons have been made in clinical trial settings using patients that are generally younger and healthier vs. patients in the RIKS-HIA register. This could partly explain the higher incidence of ischemic stroke in our study. The mean age in the PLATO-study was only 62 years vs. 70 years in the present study. Several studies have shown that age is a significant predictor of stroke following AMI [1, 2, 17–22]. Looking at other significant predictors of ischemic stroke in the multivariable Cox regression analysis, our study population differed at several critical variables. Atrial fibrillation is a well-known risk factor for ischemic stroke following AMI [2, 17–22]. The PLATO trial excluded patients requiring oral anticoagulants, thereby removing most patients with atrial fibrillation given that their treatment guidelines recommend oral anticoagulants. In our study population, atrial fibrillation was found in 14% (*n* = 6676) of all patients, with a HR of 1.92, agreeing with previous studies. Our analysis revealed that most important predictor of ischemic stroke post AMI was previous ischemic stroke (HR 3.12), as previously described [19, 21–23]. Again, when comparing our study to the PLATO-trial, the prevalence of previous ischemic stroke in our population was 6.9% (*n* = 3275) vs. 3.8% (*n* = 353) and 4.0% (*n* = 369) for the PLATO-trial ticagrelor and clopidogrel groups, respectively. Considering the large differences in prevalence of these important predictors, it can be concluded that the study population in PLATO was at lower risk compared to the population in the RIKS-HIA register regarding the risk of ischemic stroke post MI. The PLATO trial had a lower proportion of patients receiving PCI as well as statin treatment at discharge when compared to our study population. The use of statins has been found to predict a lower risk of stroke in several publications [2, 21, 22], as well as in our model. PCI during hospitalization also predicted a lower risk of ischemic stroke in the present study, as described previously [18, 19, 21, 22]. Taken together, the exclusion of patients with atrial fibrillation and the low percentage of patients with previous ischemic stroke would appear to outweigh findings that predict a higher risk of stroke in the PLATO population.

The precise mechanisms that cause ischemic stroke following AMI remain unclear. There is a widespread misconception that ischemic stroke complicating AMI is ordinarily the result of embolism from a left ventricular thrombus. However, a thrombus is almost exclusively formed after an anterior STEMI, while a large proportion of ischemic strokes occur after a non-anterior MI [24]. Moreover, one study showed that patients with diabetes mellitus had higher rates of ischemic stroke even though the proportion of patients with STEMI was lower vs. patients without diabetes mellitus [21]. Therefore, the occurrence of ischemic stroke after AMI is more likely to be related to systemic inflammation [25] and increased platelet reactivity [26] rather than to ventricular embolism. Thus, the more potent platelet inhibition manifested by ticagrelor vs. clopidogrel might be of relevance in explaining these data. A recent meta-analysis showed that, compared to aspirin, extended DAPT (> 1 year) in patients with prior MI significantly reduced cardiovascular death, recurrent MI, and stroke [27]. The PEGASUS-TIMI 54 Trial compared the long-term use of ticagrelor vs. placebo in stable patients with previous MI and demonstrated that treatment with ticagrelor significantly reduced the risk of cardiovascular death, myocardial infarction, or stroke and increased the risk of major bleeding [28]. There was no significant difference in the risk of ischemic stroke, however patients with previous ischemic stroke were excluded thus limiting the external validity. In the present study we used the number of dispensed prescriptions from a pharmacy to assess treatment duration. There was a clear trend towards longer treatment length, but the prescription number was insignificant in terms of predicting ischemic stroke in the multivariable Cox regression model. Thus we conclude that when the acute phase after MI is included, the increased use of ticagrelor is more important than treatment length *per-se* in lowering the rates of ischemic stroke. The sensitivity analysis in patients receiving at least one year of P2Y12-inhibitor treatment, also suggested a treatment effect of ticagrelor. Given a proportion of ticagrelor-usage of 52.1% in the late cohort, and the clear trend towards lower rates of ischemic stroke, we cannot rule out an underestimation of the treatment effect. In Fig. 3 the curves seem to continue to diverge more after the 12-month mark. A reduction of recurrent MI by both ticagrelor and PCI may have a preventive effect on ischemic stroke occurrence in the long term based on preservation of myocardial function. Furthermore, pleiotropic effects of ticagrelor, such as plaque stabilizing effects [29] may also be of importance. Moreover, there is a trend towards longer treatment duration in the late cohort. The further reduction of the incidence of ischemic stroke seen in the sub-analyses presented in the manuscript as well as in the Additional file 1 also supports our conclusion that an increased use of ticagrelor is associated with a lower rate of ischemic stroke in a relatively unselected AMI population when compared to clopidogrel.

### Limitations

We could not directly compare clopidogrel with ticagrelor due to selection bias (Table 4). Instead we opted to compare different time periods with different usage ratios. Our study shows that the rate of ischemic stroke after AMI is lowered following a change in preferred discharge treatment, with this rate decreasing as more patients use ticagrelor. In order to find predictors for ischemic stroke after AMI we used a multivariable Cox regression analysis for which there could have been possible confounders that were not recorded as variables in the database. The numerical method for choosing variables is also not universally favored, but in combination with relevant clinical characteristics and known risk factors available variables with potential importance were included. In our analysis we used the amount of dispensed prescriptions by a pharmacy as a proxy for treatment duration. A more detailed assessment of adherence requires a prospective design with repeated patient contacts to assess drug use. The RIKS-HIA register is validated and has been used in several previous publications, but it has certain limitations. The RIKS-HIA register is based on patients treated at a CCU. Older patients with several comorbidities are more likely to be treated outside of a CCU, creating a selection bias that excludes older and more comorbid patients limiting the external validity compared to the total AMI population both in Sweden and abroad [30]. Data from the SWEDEHEART 2014 annual report showed that when comparing patients included in the RIKS-HIA register vs. patients diagnosed with AMI in the NPR, there is a median coverage of 92% for patients <80 years, and 69% in patients ≥80 years [31]. This might lead to an underestimate in terms of assessing the risk of ischemic stroke.

## Conclusion

The introduction of ticagrelor as well as an improved management of AMI was associated with a lower rate of ischemic stroke in a relatively unselected AMI population.

## Additional file

Additional file 1: Supporting information including referenced tables and figures. (PDF 177 kb)
