# Supplementary material for: Increase in ticagrelor use over time is associated with lower rates of ischemic stroke following myocardial infarction
Source: BMC Cardiovasc Disord. 2019 Mar 4;19:51. doi: 10.1186/s12872-019-1030-6 (PMC6399852; doi:10.1186/s12872-019-1030-6)

**Supplemental material**

Increase in ticagrelor use over time is associated with lower rates of ischemic stroke following myocardial infarction

Henriksson R, Ulvenstam A, Söderström L, Mooe T.

This supplementary material has been provided for additional information.

**Supplemental methods**

We performed a number of sensitivity analyses in order to further assess our conclusion that the increased use of ticagrelor vs. clopidogrel is associated with a lower rate of ischemic stroke in unselected AMI patients.

**Ticagrelor at discharge as a variable in the multivariate cox regression model**

In order to reduce confounding caused by selection bias we compared different time periods with different use of ticagrelor rather than a direct comparison between patients treated with either clopidogrel or ticagrelor. As a sensitivity analysis we performed a multivariable Cox regression analysis using the variable "Ticagrelor at discharge" instead of the variable "Belonging to the late cohort". This was done in the final fun in the multivariable cox regression analysis. In that multivariable Cox regression model ticagrelor at discharge was associated with a significant reduction of ischemic stroke: HR 0.744 (p=0.001). See table IV.

**Matched analysis**

We also performed a matched analysis based on the most important risk factors from the Cox analysis: age, previous ischemic stroke, CABG during hospitalisation and atrial fibrillation. Based on 25262 matched patients and 455 events of ischemic stroke, the relative risk reduction of ischemic stroke in the late cohort was 35%, using Kaplan-Meier analysis. The analysis was made using "Matchit" software in the statistical package "R".

**Sub-analyses within the late cohort**

To further evaluate the impact of the switch from clopidogrel to ticagrelor in terms of the risk of ischemic stroke, we compared Kaplan-Meier estimates of the incidence of ischemic stroke in the early cohort vs. the corresponding estimates in first third of the late cohort (Supplemental material, figure I), in which 34.6% of patients used ticagrelor and 65.4% used clopidogrel. Correspondingly, in a second analysis we compared the early cohort vs. the last two thirds of the late cohort (Supplemental material, figure II). Use of ticagrelor rose to 61.1% in the last two thirds of the late cohort, with the use of clopidogrel use falling to 38.9%. Due to a shorter follow-up, and fewer patients in the subgroups of the late cohort, estimated incidence data primarily illustrate a trend towards fewer cases of ischemic stroke among patients treated with ticagrelor vs. clopidogrel at 1 year. The incidence of ischemic stroke in the early cohort was 2.8% compared to 2.5% in the first third, and 2.3% in the last two thirds of the late cohort, respectively.

**Table I** Medication at admission in the early vs. late cohort.

| | Early cohort (% (n=23447)) | Late cohort (% (n=24227)) | P-value |
|---|---|---|---|
| Aspirin | 31.6 | 30.0 | <0.001 |
| ACEI/ARB* | 31.6 | 32.8 | 0.01 |
| Statins | 25.3 | 24.8 | 0.16 |
| Beta blockers | 31.6 | 30.9 | 0.01 |
| Oral Anticoagulants | 3.3 | 3.8 | <0.01 |
| Diuretics | 20.3 | 19.0 | <0.001 |
| Calcium inhibitors | 17.7 | 18.7 | <0.01 |

* ACEI: Angiotensin converting enzyme-inhibitors; ARB: Angiotensin receptor blockers.

**Table II** Univariable predictors of ischemic stroke

| Name of predictor | HR (95% CI) | P-value |
|---|---|---|
| Age 65-74 years* | 2.20 (1.83-2.66) | <0.001 |
| Age ≥75 years | 4.26 (3.6-5.03) | <0.001 |
| Female sex | 1.38 (1.23-1.55) | <0.001 |
| Hypertension | 1.98(1.75-2.24) | <0.001 |
| Diabetes | 1.60(1.42-1.81) | <0.001 |
| STEMI† | 0.93 (0.82-1.05) | 0.24 |
| Atrial fibrillation | 3.05 (2.7-3.44) | <0.001 |
| Heart failure during hospitalisation | 2.21 (1.96-2.5) | <0.001 |
| Smoking | 0.62 (0.53-0.72) | <0.001 |
| Previous PCI‡ | 1.04 (0.85-1.27) | 0.73 |
| Previous MI§ | 1.75 (1.47-2.07) | <0.001 |
| Previous dialysis | 1.95 (1.08-3.53) | 0.03 |
| Previous PAD** | 2 (1.63-2.46) | <0.001 |
| Previous ischemic stroke | 5.20 (4.6-5.95) | <0.001 |
| Previous hemorrhagic stroke | 2.69 (1.93-3.75) | <0.001 |
| Thrombolysis during hospitalisation | 0.91 (0.56-1.50) | 0.72 |
| PCI during hospitalisation | 0.46 (0.41-0.51) | <0.001 |
| CABG during hospitalisation†† | 1.73 (1.17-2.58) | 0.01 |
| Aspirin at discharge | 0.46 (0.37-0.57) | <0.001 |
| ACEI/ARB at discharge‡‡ | 0.90 (0.79-1.03) | 0.13 |
| Statins at discharge | 0.41 (0.35-0.47) | <0.001 |
| Oral Anticoagulants at discharge | 1.7 (1.39-2.09) | <0.001 |
| Beta-blockers at discharge | 0.86 (0.72-1.03) | 0.11 |
| Calcium inhibitors at discharge | 1.28 (1.11-1.48) | <0.01 |
| Dispensed prescriptions: 2 | 0.8 (0.63-0.958) | 0.02 |
| Dispensed prescriptions: 3 | 0.96 (0.75-1.23) | 0.76 |
| Dispensed prescriptions: 4 | 0.62 (0.54-0.72) | <0.001 |
| Diuretics at discharge | 2.08 (1.85-2.33) | <0.001 |
| eGFR§§ | 0.98 (0.98-0.98) | <0.001 |

* Age ≤64 years was used as the reference age for different categories.

† ST-elevation myocardial infarction
‡ Percutaneous coronary intervention
§ Myocardial infarction
** Peripheral artery disease
†† Coronary artery bypass graft surgery
‡‡ ACEI: Angiotensin converting enzyme-inhibitors; ARB: Angiotensin receptor blockers
§§ Estimated glomerular filtration rate

**Table III** First run of the multivariable Cox regression model.

| Predictor Variable | HR (95% Confidence Interval) | P-value |
|---|---|---|
| Age 65-74 years* | 1.8 (1.43-2.28) | <0.001 |
| Age ≥75 years | 2.53 (1.99-3.22) | <0.001 |
| Female sex | 1.09 (0.95-1.26) | 0.21 |
| Hypertension | 1.19 (1.01-1.39) | 0.04 |
| Smoking | 1.07 (0.88-1.3) | 0.5 |
| Diabetes | 1.18 (1.01-1.38) | 0.04 |
| STEMI† | 1.21 (1.04-1.4) | 0.02 |
| Atrial fibrillation | 1.86 (1.58-2.19) | <0.001 |
| Heart failure during hospitalisation | 1.23 (1.05-1.44) | <0.01 |
| PCI during hospitalisation‡ | 0.77 (0.65-0.9) | <0.01 |
| CABG during hospitalisation§ | 1.44 (0.72-2.91) | 0.31 |
| Previous myocardial infarction | 0.93 (0.75-1.16) | 0.52 |
| Previous ischemic stroke | 3.19 (2.7-3.77) | <0.001 |
| Previous hemorrhagic stroke | 1.36 (0.88-2.12) | 0.16 |
| Previous peripheral artery disease | 1.18 (0.92-1.53) | 0.19 |
| Previous dialysis | 1.71 (0.83-3.51) | 0.15 |
| Statins at discharge | 0.84 (0.69-1.02) | 0.08 |
| Oral anticoagulants at discharge | 0.88 (0.67-1.17) | 0.38 |
| Aspirin at discharge | 0.8 (0.59-1.07) | 0.13 |
| Calcium inhibitors at discharge | 0.95 (0.8-1.4) | 0.6 |
| Diuretics at discharge | 1.05 (0.89-1.23) | 0.57 |
| Belonging to the late cohort | 0.84 (0.73-0.96) | 0.01 |
| Dispensed prescriptions: 2 | 0.85 (0.67-1.07) | 0.16 |
| Dispensed prescriptions: 3 | 1.11 (0.86-1.44) | 0.42 |
| Dispensed prescriptions: 4 | 0.86 (0.73-1.01) | 0.07 |
| eGFR** | 0.997 (0.994-1) | 0.18 |

* Age ≤64 years was used as the reference age for different categories.

† ST-elevation myocardial infarction
‡ Percutaneous coronary intervention
§ Coronary artery bypass graft surgery
** Estimated glomerular filtration rate

**Table IV** Final run of the multivariable cox regression analysis using ticagrelor at discharge as a variable instead of belonging to the late cohort

| Predictor Variable | HR (95% Confidence Interval) | P-value |
|---|---|---|
| Age 65-74 years[*] | 1.64 (1.36-2.02) | <0.001 |
| Age ≥75 years | 2.14 (1.74-2.63) | <0.001 |
| Hypertension | 1.17 (1.02-1.34) | 0.03 |
| Diabetes | 1.23 (1.07-1.41) | 0.01 |
| STEMI[†] | 1.29 (1.13-1.48) | <0.001 |
| Atrial fibrillation | 1.89 (1.65-2.17) | <0.001 |
| Heart failure during hospitalisation | 1.29 (1.13-1.48) | <0.001 |
| PCI during hospitalisation[‡] | 0.74 (0.65-0.86) | <0.001 |
| CABG during hospitalisation[§] | 1.93 (1.28-2.9) | <0.01 |
| Previous ischemic stroke | 3.1 (2.68-3.59) | <0.001 |
| Previous hemorrhagic stroke | 1.51 (1.06-2.17) | 0.02 |
| Statins at discharge | 0.8 (0.6-0.95) | 0.01 |
| Ticagrelor at discharge | 0.744 (0.63-0.86) | 0.001 |
| eGFR[**] | 0.996 (0.993-0.999) | 0.01 |

[*] Age ≤64 years was used as the reference age for different categories.

[†] ST-elevation myocardial infarction
[‡] Percutaneous coronary intervention
[§] Coronary artery bypass graft surgery
[**] Estimated glomerular filtration rate

**Figure I** Kaplan-Meier estimates of the incidence of ischemic stroke in the early cohort vs. the first third of the late cohort

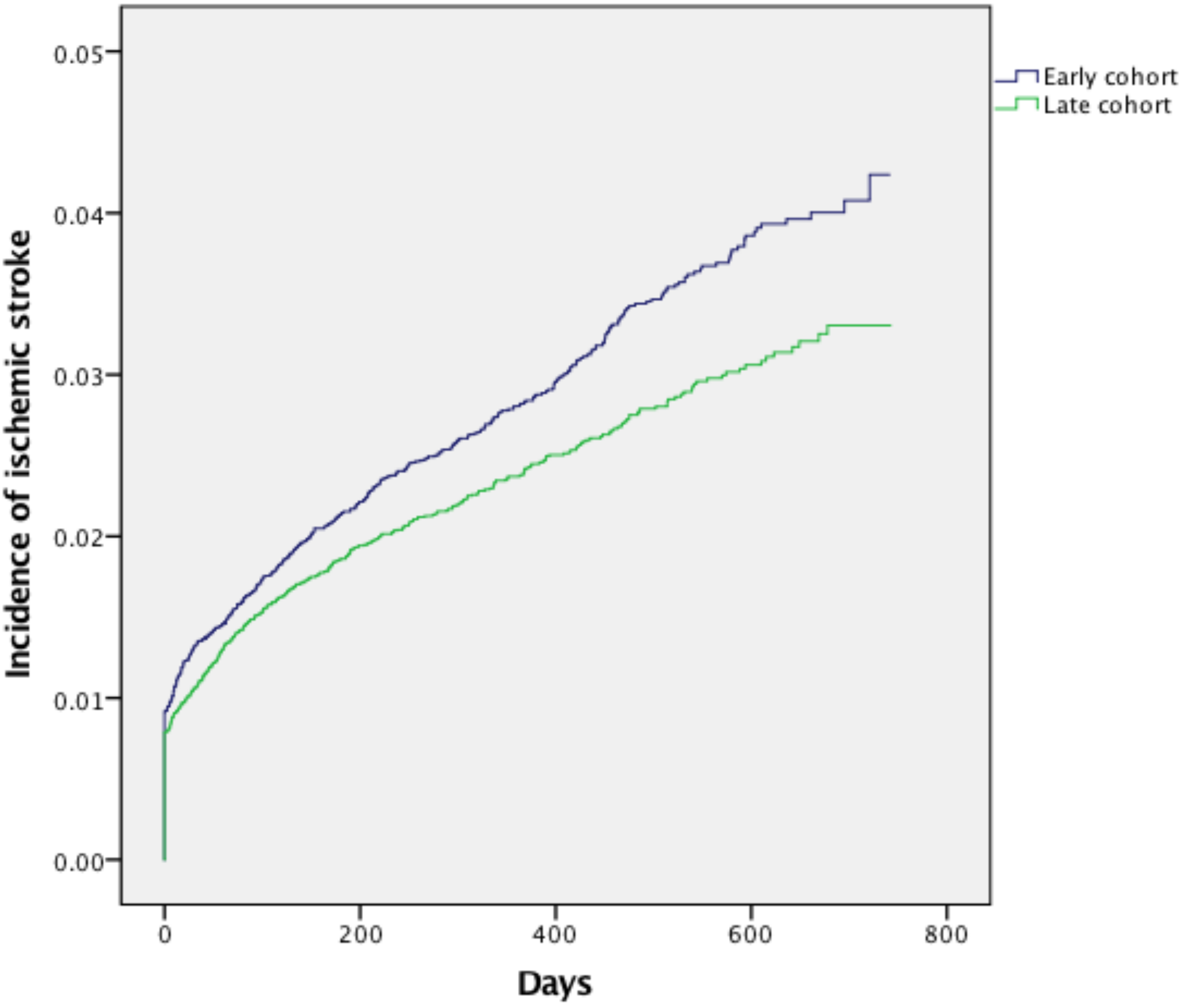

**Figure II** Kaplan-Meier estimates of the incidence of ischemic stroke in the early cohort vs. the last two thirds of the late cohort

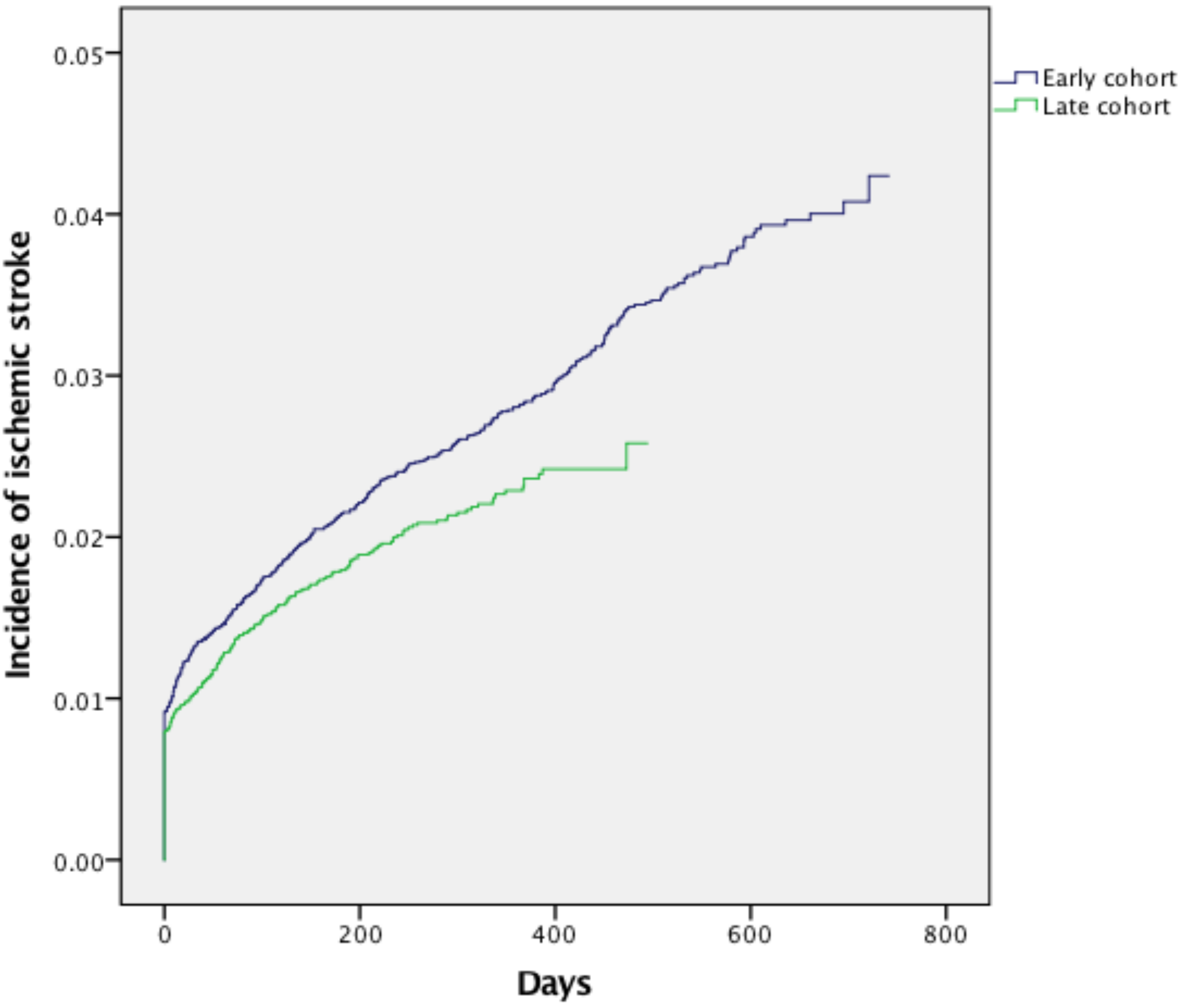

Supplement: Supplementary file 1 — Supporting information including referenced tables and figures. (PDF 177 kb) [file 12872_2019_1030_MOESM1_ESM.pdf]
